# Multi-component gene network design as a survival strategy in diverse environments

**DOI:** 10.1186/s12918-018-0609-3

**Published:** 2018-09-26

**Authors:** Xinyue Luo, Ruijie Song, Murat Acar

**Affiliations:** 1 Department of Molecular Cellular and Developmental Biology, Yale University, 219 Prospect Street, New Haven, CT 06511 USA; 2 Systems Biology Institute, Yale University, 850 West Campus Drive, Room 122, West Haven, CT 06516 USA; 3 Interdepartmental Program in Computational Biology and Bioinformatics, Yale University, 300 George Street, Suite 501, New Haven, CT 06511 USA; 4 Department of Physics, Yale University, 217 Prospect Street, New Haven, CT 06511 USA

**Keywords:** Gene network, Cell-environment interaction, Systems biology, Evolution, Cell, Survival, Galactose network, Yeast

## Abstract

**Background:**

Gene-environment interactions are often mediated though gene networks in which gene expression products interact with other network components to dictate network activity levels, which in turn determines the fitness of the host cell in specific environments. Even though a gene network is the right context for studying gene-environment interactions, we have little understanding on how systematic genetic perturbations affects fitness in the context of a gene network.

**Results:**

Here we examine the effect of combinatorial gene dosage alterations on gene network activity and cellular fitness. Using the galactose utilization pathway as a model network in diploid yeast, we reduce the copy number of four regulatory genes (*GAL2*, *GAL3*, *GAL4*, *GAL80*) from two to one, and measure the activity of the perturbed networks. We integrate these results with competitive fitness measurements made in six different rationally-designed environments containing different galactose concentrations representing the natural induction spectrum of the galactose network. In the lowest galactose environment, we find a nonlinear relationship between gene expression and fitness while high galactose environments lead to a linear relationship between the two with a saturation regime reached at a sufficiently high galactose concentration. We further uncover environment-specific relevance of the different network components for dictating the relationship between the network activity and organismal fitness, indicating that none of the network components are redundant.

**Conclusions:**

These results provide experimental support to the hypothesis that dynamic changes in the environment throughout natural evolution is key to structuring natural gene networks in a multi-component fashion, which robustly provides protection against population extinction in different environments.

**Electronic supplementary material:**

The online version of this article (10.1186/s12918-018-0609-3) contains supplementary material, which is available to authorized users.

## Background

Cells are extraordinarily complex systems that emerged out of billions of years of evolution that morphed the earliest prokaryotes into the diverse universe of biological organisms today. Having undergone such a lengthy optimization process, the parameters of these systems, including the expression levels of the genes in the cell, are surely near their optimal values for fitness. It follows that, as a general matter, significant changes to such parameters will likely cause a reduction in fitness, and indeed halving the dosage of most essential genes in diploid yeast has been shown to give rise to negative fitness impact, especially for genes of which the protein products interact with other proteins [1]. Similarly, in humans, duplications increasing the dosage of critical genes are known to cause severe genetic diseases [2]. Such drastic dosage changes aside, the amount of the product of a gene in the cell is hardly constant over time, due to the inherent stochasticity, or noise, of the transcription and translation processes as well as other parts of the cellular machinery [3–9], resulting in a change in the effective dosage of the gene.

However, genes generally do not interact with the environment in isolation. Instead, those interactions typically occur through the action of often complex gene networks: the environment interacts with specific gene expression products, which alter the activity level of the network through their interactions with other network components. That activity level then determines the cell’s fitness in that environment. To study the effect of genetic perturbations without considering it in the context of the gene network is not unlike the parable of the blind men and an elephant: it misses the forest for the trees.

Work has been done on changing the dosage of individual genes [1, 10, 11], as well as changes in the dosage of entire gene networks, which, like the dosage of individual genes, can occur due to changes in cell volume and genome content during cell cycle [12–14]. In the latter case, a molecular mechanism intrinsic to the gene network topologies has been discovered [12] and multiple instances of such topologies have been found in the budding yeast genome [13]. Nonetheless, little has been done to study the effect of dosage perturbations of individual genes in a gene network context. A comprehensive understanding on the links between gene dosage perturbation, network activity levels and fitness in the context of a natural gene network would provide important insights into why certain gene expression levels and network topologies have been selected over evolutionary timescales.

## Results

### Systematically characterizing dosage-fitness relationships in the yeast GAL network

**Fig. 1:**
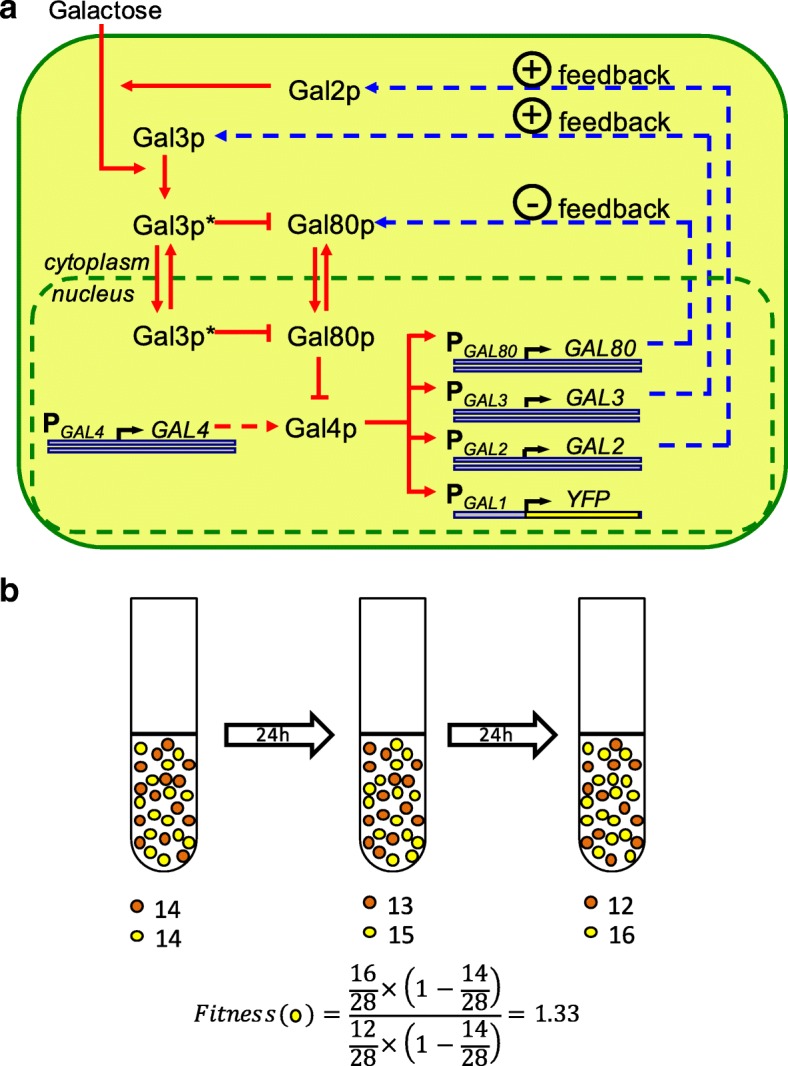
**a** Network architecture built by the GAL2, GAL3, GAL4, and GAL80 regulatory genes. The red lines denote the four-stage signaling cascade. The galactose-bound state of Gal3p is denoted by Gal3p*. Pointed and blunt red arrows reflect activation and inhibition, respectively. The dashed blue arrows denote feedback loops established by Gal2p, Gal3p and Gal80p. The dashed red arrow represents the constitutive expression of the Gal4 proteins. The double red arrows denote translocation of Gal80p and Gal3p* between cytoplasm and nucleus. **b** Illustration of the fitness metric. The strain under investigation is mixed with a reference strain, allowed to equilibrate and then grown for 48 h. The fitness value is calculated from the population ratio of the two strains at the beginning and end of the growth period as shown

For a systematic characterization of dosage-fitness relationships, we used the *Saccharomyces cerevisiae* galactose utilization (GAL) network as the model system, as it is arguably the most well-characterized gene network in eukaryotic biology (Fig. 1a) [7, 12, 15–19]. Transcription of genes in the GAL network is controlled by the constitutively expressed transcription factor Gal4p, whose activity in the absence of galactose is inhibited by the inhibitor Gal80p. The signal transducer Gal3p is activated by galactose and binds to the inhibitor Gal80p, relieving Gal4p from Gal80p’s suppression to activate all GAL network genes except for Gal4p itself [20–23]. Gal2p is a galactose permease that imports extracellular galactose molecules into the cell [24], and thus may potentially impact both the level of GAL network activation and the benefit the cells receive from the production of GAL network proteins.

**Fig. 2:**
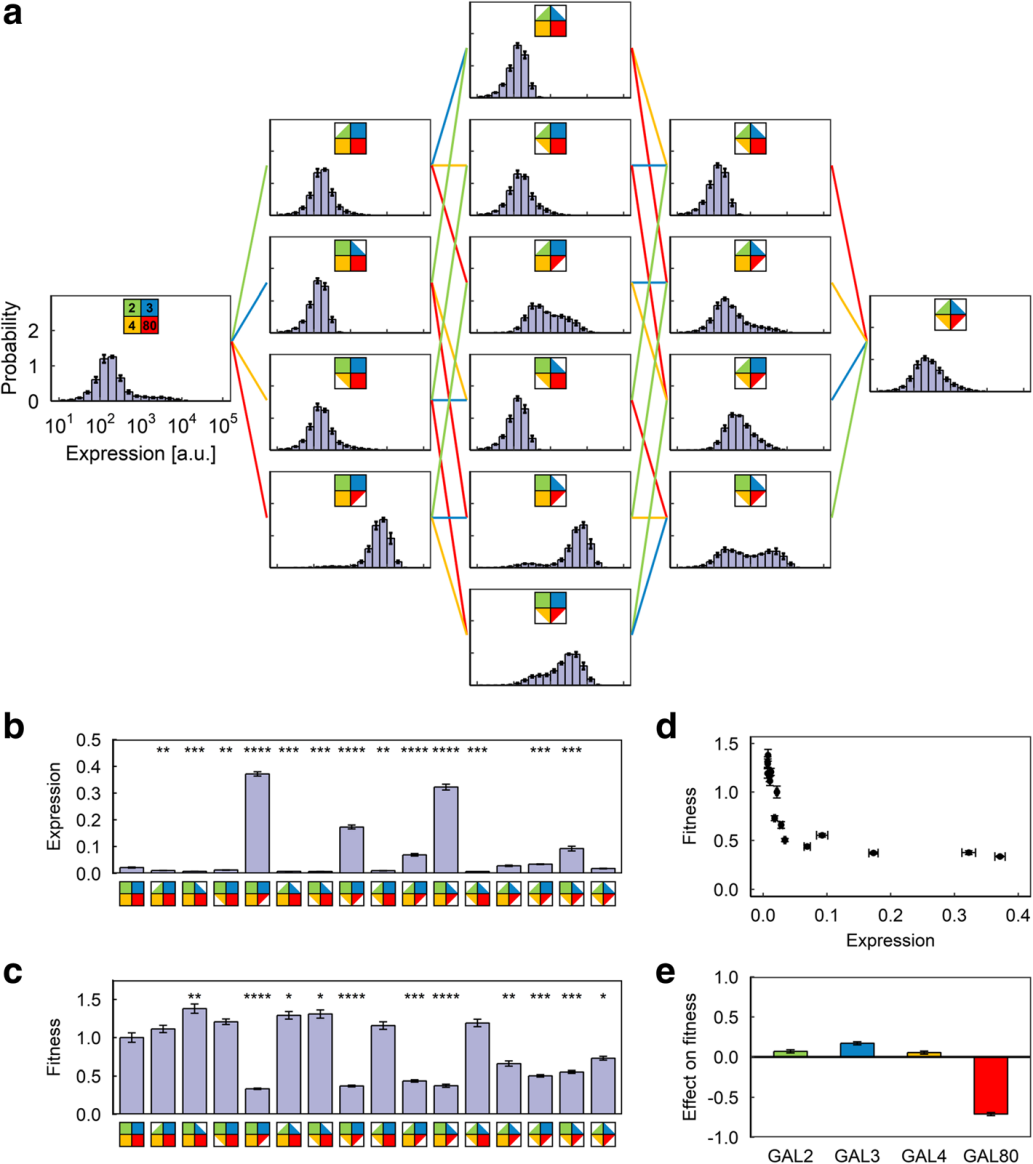
Results of the competition experiment in Environment A (0.03% galactose, 0.1% mannose). **a** Final expression level distributions of the 16 strains. **b** Average P_*GAL1*_-YFP expression level of the 16 strains. Expression levels are normalized to the expression level of the reference strain in the same sample. Error bars indicate s.e.m. (*N* = 9). Stars indicate statistically significant differences from wild-type strain as determined by a two-tailed Student’s t-test (Bonferroni-corrected *p*-value: ****: *p* < 0.0001; ***: *p* < 0.001; **: *p* < 0.01; *: *p* < 0.05). **c** Average fitness value of the 16 strains, normalized to the average fitness value of the wild-type strain. Error bars indicate s.e.m. (*N* = 9). Stars indicate statistically significant differences from wild-type strain as determined by a two-tailed Student’s t-test (Bonferroni-corrected p-value: ****: *p* < 0.0001; ***: *p* < 0.001; **: *p* < 0.01; *: *p* < 0.05). **d** Plot of expression vs. fitness for the 16 strains. Error bars indicate s.e.m. (*N* = 9). **e** Average effect of copy number reduction on fitness for the four genes in this environment. Error bars indicate uncertainty calculated from the s.e.m. of the fitness measurements

Using P_*GAL1*_-YFP as our single-cell level reporter of the GAL network activity, we constructed 16 different diploid yeast strains by combinatorially reducing the copy number of the four regulatory genes of interest (GAL2, GAL3, GAL4, GAL80) from two to one (Fig. 2a). We confirmed that these strains have no GAL network activity when grown in 2% glucose (Additional file 1: Figure S1). We next measured the comparative fitness and YFP expression levels in these strains in three kinds of environments designed to represent a diverse set of environmental conditions: an environment with minimal (0.03%) galactose and a moderate amount (0.1%) of mannose (a non-inducing neutral carbon source for the GAL network); a “mirror” environment with moderate (0.1%) galactose and minimal (0.03%) mannose; and several pure-galactose environments with galactose concentrations ranging from 0.1 to 2%. To measure the fitness of a strain in a given environment, we mixed it with cells from a reference strain carrying the constitutively expressed P_*TEF1*_-mCherry reporter in the diploid wild-type background, grew the mixture (after appropriate equilibration) for 48 h, and calculated its fitness [25] relative to the reference strain from the ratio of cells at the beginning and end of the growth period (Fig. 1b). We confirmed that the fitness value measured is invariant with respect to the ratio of cells at the beginning of the growth period (Additional file 1: Figure S2).

### Dosage-fitness relationships in mixed sugar environments

In the first environment, with minimal galactose and moderate neutral sugar, we observed significant differences in both expression level and the fitness of the 16 strains (Fig. 2). Most strikingly, reducing the dosage of GAL80 significantly increased the expression level and at the same time caused significant loss of fitness (Fig. 2b and c), while reducing the dosage of GAL3 has a much smaller positive impact on fitness (Fig. 2e). However, as can be seen from the hockey-stick-like plot of fitness against expression, the exact amount of expression level increase had a nonlinear relationship with the fitness lost (Fig. 2d), and indeed strains whose average expression level differ by up to 10-fold displayed similar levels of fitness, indicating that the fitness cost is linked to the very act of activating the GAL network genes, rather than simply the energy cost of producing excess GAL network proteins.

**Fig. 3:**
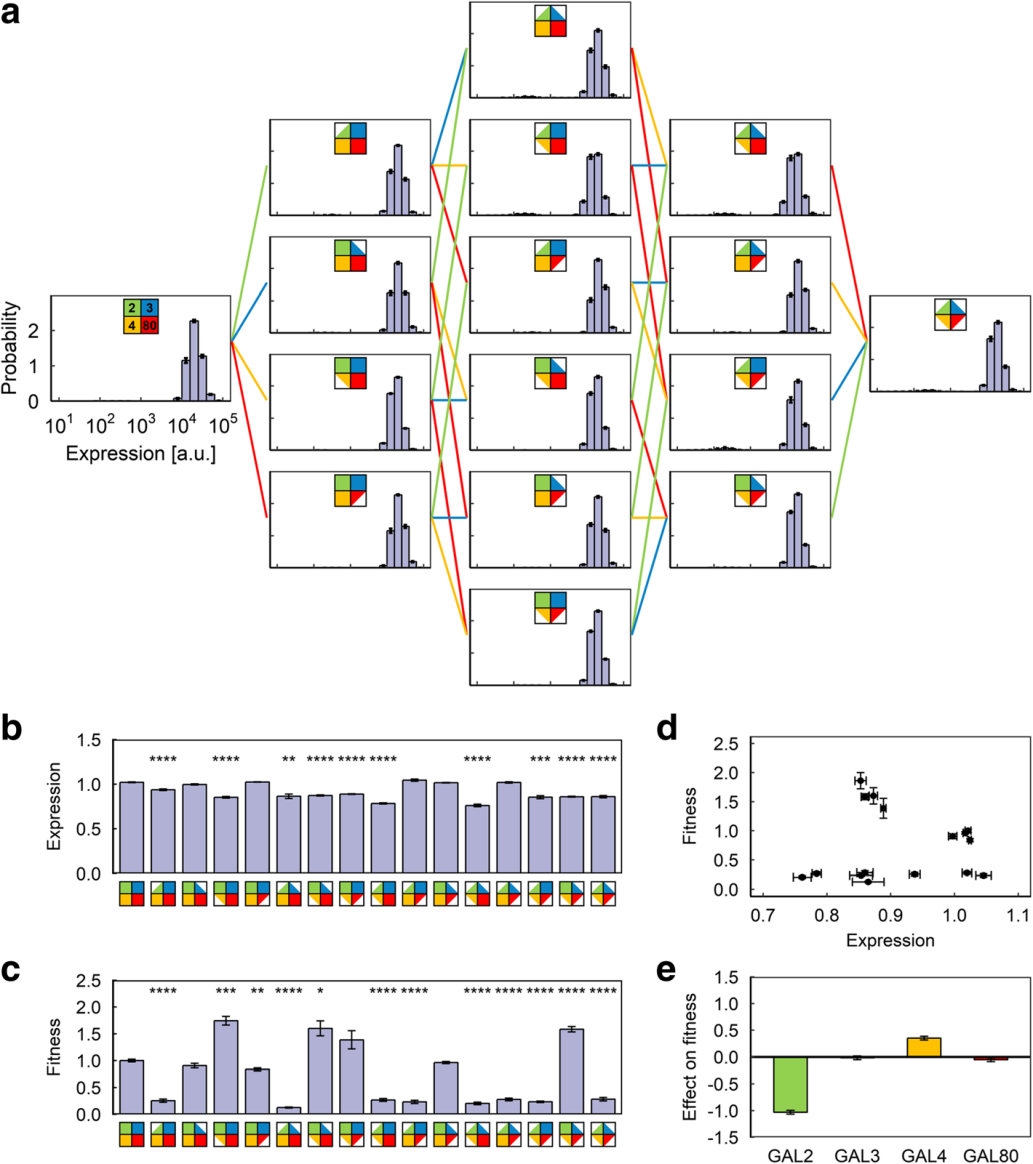
Results of the competition experiment in Environment B (0.1% galactose, 0.03% mannose). **a** Final expression level distributions of the 16 strains. **b** Average P_*GAL1*_-YFP expression level of the 16 strains. Expression levels are normalized to the expression level of the reference strain in the same sample. Error bars indicate s.e.m. (*N* = 9). Stars indicate statistically significant differences from wild-type strain as determined by a two-tailed Student’s t-test (Bonferroni-corrected *p*-value: ****: *p* < 0.0001; ***: *p* < 0.001; **: *p* < 0.01; *: *p* < 0.05). **c** Average fitness value of the 16 strains, normalized to the average fitness value of the wild-type strain. Error bars indicate s.e.m. (*N* = 9). Stars indicate statistically significant differences from wild-type strain as determined by a two-tailed Student’s t-test (Bonferroni-corrected p-value: ****: *p* < 0.0001; ***: *p* < 0.001; **: *p* < 0.01; *: *p* < 0.05). **d** Plot of expression vs. fitness for the 16 strains. **e** Average effect of copy number reduction on fitness for the four genes in this environment. Error bars indicate uncertainty calculated from the s.e.m. of the fitness measurements

We then assessed the results obtained from the second environment (Fig. 3), which is a mirror of the first: minimal neutral sugar (0.03%) and moderate galactose (0.1%). Here we observed two separate trends: strains with the GAL2 copy number halved displayed a significant loss of fitness (Fig. 3c, e), despite having similar levels of GAL network expression (Fig. 3b), which we interpreted to be due to their reduced ability to internalize the environmental galactose. While other hexose transporters capable of transporting galactose do exist in yeast, it is apparent that at least in this environment, they are unable to make up for the reduction in Gal2p expression in these strains, probably because they have less affinity for galactose and may require a higher concentration to function. Meanwhile, of the strains that have two copies of GAL2, there’s a clear inverse correlation between the expression level and fitness, indicating that at 0.1% galactose, the cost of expressing additional GAL network proteins outweighs the benefit (Fig. 3d).

### Dosage-fitness relationship in pure galactose environments

**Fig. 4:**
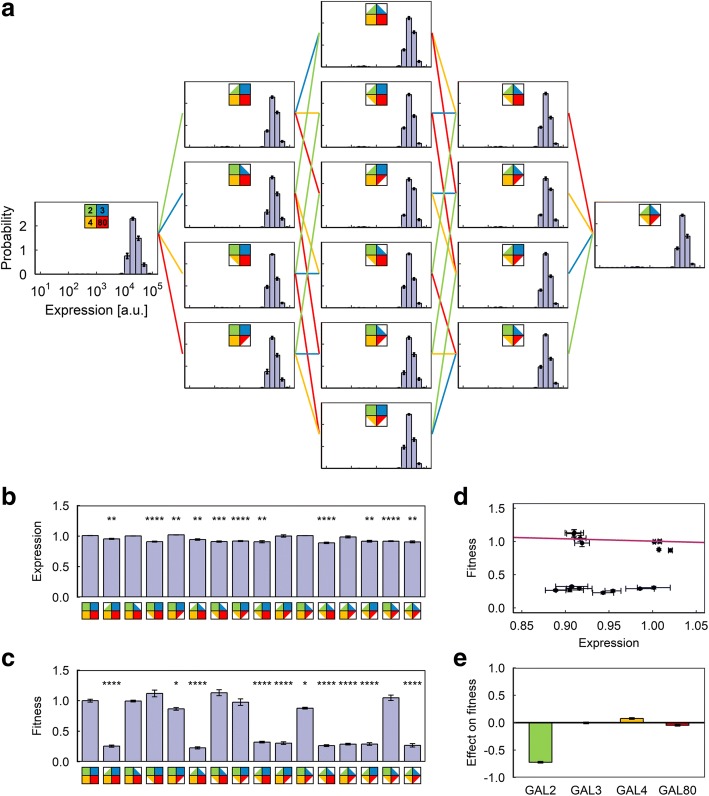
Results of the competition experiment in Environment C (0.1% galactose). **a** Final expression level distributions of the 16 strains. **b** Average P_*GAL1*_-YFP expression level of the 16 strains. Expression levels are normalized to the expression level of the reference strain in the same sample. Error bars indicate s.e.m. (N = 9). Stars indicate statistically significant differences from wild-type strain as determined by a two-tailed Student’s t-test (Bonferroni-corrected p-value: ****: *p* < 0.0001; ***: *p* < 0.001; **: *p* < 0.01; *: *p* < 0.05). **c** Average fitness value of the 16 strains, normalized to the average fitness value of the wild-type strain. Error bars indicate s.e.m. (N = 9). Stars indicate statistically significant differences from wild-type strain as determined by a two-tailed Student’s t-test (Bonferroni-corrected p-value: ****: *p* < 0.0001; ***: *p* < 0.001; **: *p* < 0.01; *: *p* < 0.05). **d** Plot of expression vs. fitness for the 16 strains. Solid line is the prediction of the fitted linear model. Error bars indicate s.e.m. (N = 9). **e** Average effect of copy number reduction on fitness for the four genes in this environment. Error bars indicate uncertainty calculated from the s.e.m. of the fitness measurements

Since activating the GAL network carries significant fitness cost and the presence of the neutral sugar permitted some cells to survive without activating the network (Fig. 3a), we decided to examine the behavior of these strains when the neutral sugar is removed and only 0.1% galactose is present in the environment (Fig. 4). All cells in this environment necessarily pay the cost associated with activating the GAL network, eliminating the confounding factor and allowing for a clearer evaluation of the relationship between additional expression and fitness. As we expected, the GAL2-dependent fitness reduction also appeared in this environment (Fig. 4c, e). However, unlike in the previous environment (Fig. 3e), reducing the copy number of GAL4 only has a minimal effect on fitness (Fig. 4e). This is consistent with these cells’ inability to avoid activating the network (and the associated fitness cost). Indeed, the amount of fitness lost due to increased expression is significantly smaller (Fig. 4c-d) compared to the previous environment (Fig. 3d).

**Fig. 5:**
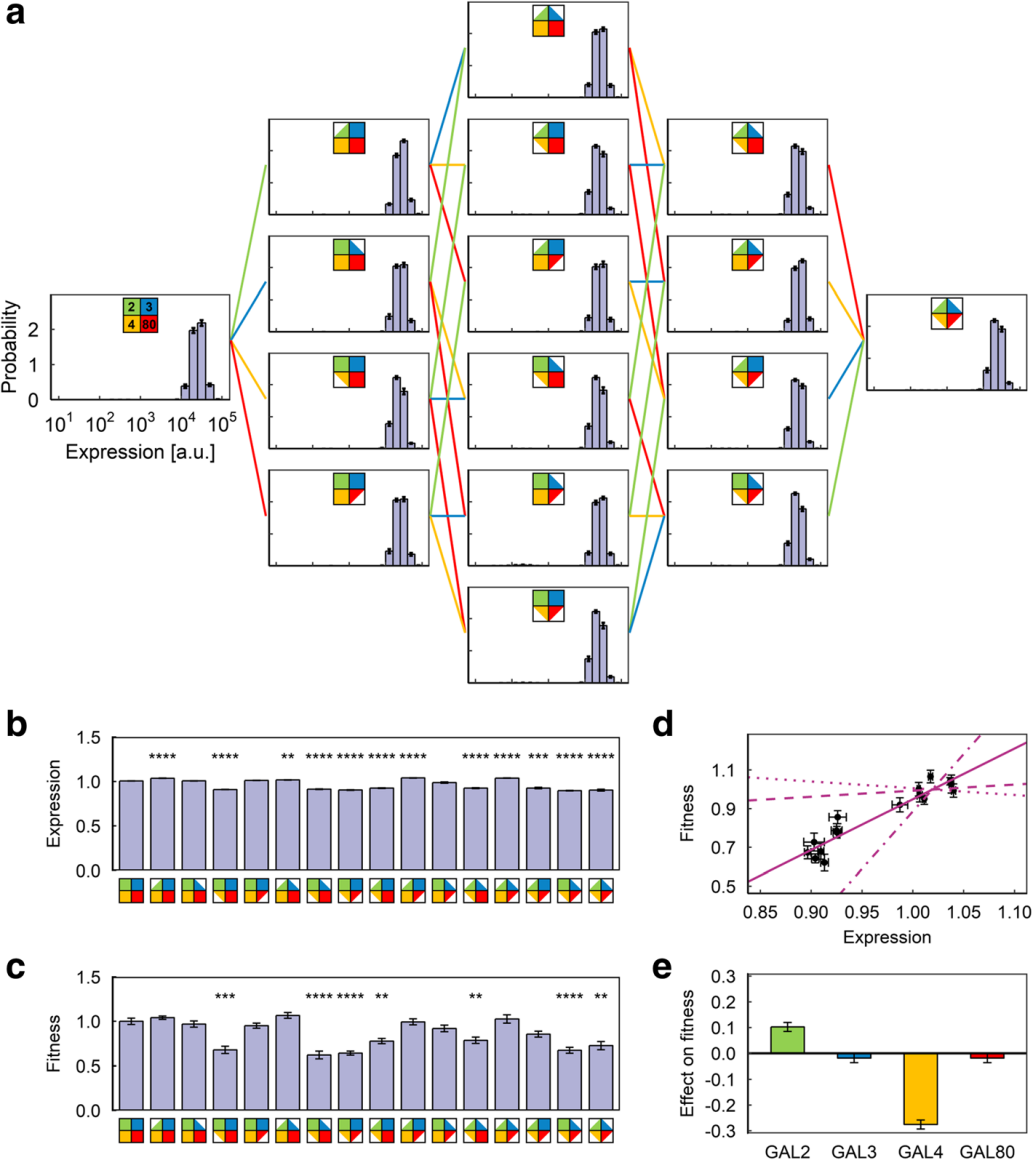
Results of the competition experiment in Environment D (1% galactose). **a** Final expression level distributions of the 16 strains. **b** Average P_*GAL1*_-YFP expression level of the 16 strains. Expression levels are normalized to the expression level of the reference strain in the same sample. Error bars indicate s.e.m. (N = 9). Stars indicate statistically significant differences from wild-type strain as determined by a two-tailed Student’s t-test (Bonferroni-corrected p-value: ****: *p* < 0.0001; ***: *p* < 0.001; **: *p* < 0.01; *: *p* < 0.05). **c** Average fitness value of the 16 strains, normalized to the average fitness value of the wild-type strain. Error bars indicate s.e.m. (N = 9). Stars indicate statistically significant differences from wild-type strain as determined by a two-tailed Student’s t-test (Bonferroni-corrected p-value: ****: *p* < 0.0001; ***: *p* < 0.001; **: *p* < 0.01; *: *p* < 0.05). **d** Plot of expression vs. fitness for the 16 strains. Solid line is the prediction of the fitted linear model. The dotted, dashed, and dotted-dashed lines show the prediction of the model at 0.1%, 0.3% and 2% galactose, respectively. Error bars indicate s.e.m. (N = 9). **e** Average effect of copy number reduction on fitness for the four genes in this environment. Error bars indicate uncertainty calculated from the s.e.m. of the fitness measurements

We then decided to explore the impact of further increasing the environmental galactose concentration in pure-galactose environments with 0.3%, 1% or 2% galactose. Unlike the 0.1% galactose environment, reducing GAL2 dosage turned out to either have no significant effect on fitness (Additional file 1: Figure S3E) or slightly increase fitness (Fig. 4e; Additional file 1, Figure S4E) in these higher galactose environments, even though the activity of the network, as indicated by the expression level of the reporter, did not change significantly (Fig. 5a; Additional file 1: Figures. S3A, S4A) compared to the 0.1% galactose case (Fig. 4a), and so the cells are expressing similar amounts of Gal2p. We interpret the absence of fitness reduction to be due to the non-specific hexose transporters [24, 26] such as Hxt4p [27] being able to assist in galactose import at these higher galactose concentrations. The slight increase in fitness at 1% and 2% galactose is consistent with previous observations that GAL2 overexpression can decrease uptake of galactose [28] and reduce growth rate [29]. Meanwhile, the benefit from being able to metabolize additional galactose begins to overtake the cost of producing additional GAL network proteins as the environmental galactose concentration increases. As a result, we see that the strains with one copy of the *GAL4* gene (the constitutively-expressed master transcription factor of the entire GAL network) gradually lose ground to the wild type in terms of fitness as the galactose concentration rises: at 0.3% galactose, the expression level is approximately fitness-neutral (Additional file 1: Figure S3D-E), while at even higher galactose levels (1% or 2%) the higher expression level is associated with an increase in fitness (Fig. 5d-e; Additional file 1: Figure S4D-E).

To obtain another layer of quantitative understanding about the relationship between expression level, galactose concentration and fitness, we fitted the expression level and fitness data from the 0.3% and 1% galactose-only environments to a simple linear model (Fig. 5d; Additional file 1: Figure S3D). The model provided a reasonably good match between expression level and fitness in the 0.1% galactose environment for strains with two copies of GAL2 (Fig. 4d, solid line), but predicted a significantly steeper curve than experimentally observed for the 2% galactose environment (Additional file 1: Figure S4D, solid line), indicating saturation. Indeed, the experimental results obtained in the 2% galactose environment were far closer to what the model predicts for the 1% galactose environment (Additional file 1, Figure S4D: dotted line).

### Epistasis analysis

Finally, we examined the epistatic effects of dosage reduction in multiple genes. In each environment, we first computed the overall epistatic deviation for the eleven strains with dosage reductions in at least two genes (Additional file 1: Figure S5, left column). We found significant epistatic deviations at α = 0.05 for five dosage-reduction combinations in environments A and B, two combinations in environment C, and one combination in environment D. We next computed higher-order epistatic deviations and found only three combinations with significant higher-order epistatic interactions, all in environment A (Additional file 1: Figure S5, right column). We attribute the absence of additional cases of higher-order epistatic deviations to the high level of uncertainty arising out of error propagation: each higher-order epistatic deviation measure is calculated from at least seven fitness measurements.

## Discussion

Our observations provide new insights into how gene dosage alterations influence cellular fitness. The fitness levels of most strains with gene dosage alterations were found to be environment-dependent, and reducing the copy number of GAL2, GAL4 or GAL80 caused substantial reductions in fitness in at least of one of the environments used in our study. The “quadruple deletion” strain in which the copy number of all four genes have been halved is less fit than the wild-type strain in five of the six environments tested and have similar fitness in the remaining one (environment E). We note that, while network dosage compensation [12–14] keeps the activity of the GAL network promoters similar between the wild type and the quadruple-deletion strain, having one vs two genes still makes a difference in overall gene expression from the specific genes.

We note that halving the dosage of the genes may not reduce their expression level exactly by half, with the exception of the constitutively expressed transcription factor GAL4 [30]. The remaining genes are all regulated by the GAL network machinery with its multiple feedback loops that can potentially either mitigate or amplify the effect of dosage reduction on network output. While the focus of this study has been to uncover how gene dosage variations affect network output and fitness of the host cells, the shifts we observed in downstream GAL1 promoter activity and fitness clearly indicate that expression level changes have taken place as a result of the gene dosage alterations.

Our results lend empirical support to our original hypothesis that the expression levels in wild-type cells were sufficiently optimized such that perturbations are to be detrimental. Regarding GAL3, simply reducing its copy number in our experiments either has no effect on fitness or a slightly positive one, depending on the environment. However, this is not the entire picture. The GAL network has a second inducer: the galactokinase Gal1p also serves as a weak, but highly expressed, inducer [20, 22, 31] of the GAL network. To avoid the confounding factor through galactose metabolism and its effect on overall fitness, we did not perturb the GAL1 dosage in this study. Also, due to experimental limitations, we were only able to reliably measure fitness after cells have adapted to the environment and reached steady state. While Gal3p’s role as the activator of the GAL network after the network’s initial activation can be filled by Gal1p, Gal3p still plays an essential role in the initial activation of the network, as the basal expression of Gal1p in the absence of galactose is virtually nonexistent [32]. Indeed, complete deletion of *GAL3* is known to dramatically increase the induction lag from a few minutes to several days [33, 34]. Thus, while reducing the dosage of *GAL3* might increase the fitness after the cells have adapted to its environment, it almost certainly will negatively impact the ability of the cells to react and adapt to increases in environmental galactose.

Genes typically interact with the environment through the activity of gene networks, and it is not possible to obtain a comprehensive understanding of the impact of genetic perturbations on cellular fitness without considering the complete picture in the context of a gene network. By systematically perturbing the dosage of the *GAL* network regulatory genes and studying the impact of each perturbation on the network activity level and fitness of the cell, we demonstrate the role played by each network component in determining the relationship between the network activity and organismal fitness in specific environments, with the combined effects of the network genes ensuring that the organism is competitive in a wide range of environments. Our results provide experimental support to the hypothesis that selection for multi-component gene network structures is a beneficial evolutionary strategy, with each component’s expression taking the lead in fitness optimization in a specific environment, as such a strategy can facilitate robust protection against population extinction in changing environments.

## Methods

### Construction of yeast strains

All *S. cerevisiae* strains used in this study have the W303 genetic background. Complete descriptions of all strains can be found in Additional file 1: Table S1.

We constructed the 16 different diploid yeast strains carrying one or two copies of each of the four GAL regulatory genes by mating haploid strains of the opposite mating types that have zero, one or two knockouts of the four genes. We amplified either the *P*_*AgTEF*_*-natNT2-tADH1* cassette from pYM17 (Euroscarf) or *P*_*TEF*_*-kanMX4-tTEF* cassette from pRS400 (Boekes Lab, NYU) with primers carrying 60 bp homology to the immediate upstream and downstream of target gene to knock out its entire open reading frame. Specifically, *GAL2* or/and *GAL3* were knocked out from the haploid strain MAURA3-YFP of mating type MATα, while *GAL4* or/and *GAL80* were knocked out from the haploid strain MALEU2 of mating type MATa. To obtain MAURA3-YFP, we first PCR-amplified the *HIS5-P*_*GAL1*_*-YFP-tCYC1* cassette from the plasmid pHIS5G1YFP using primers carrying 60 bp homology to the *ho* locus and transformed the PCR-amplified linear DNA into the strain MA0001, which is a haploid wild-type strain with MATα background. Then, the plasmid pRS306 linearized at BstBI cut site within *URA3* was transformed into the resulting strain at the *ura* locus. The strain MALEU2 was constructed by inserting linearized plasmid pRS305 (cut at the AflII site within *LEU2*) into MA0002, which is a wild-type haploid strain with MATa background. The competing strains XLUYLmC and XLUYLmCdd80 were constructed in a similar manner. XLUYLmC was obtained by mating MAURA3-YFP with XLLHmC, which has a single copy of *HIS5-P*_*TEF1*_*-mCherry-tCYC1* cassette integrated in the *ho* locus of MALEU2. The *HIS5-P*_*TEF1*_*-mCherry-tCYC1* cassette was constructed by replacing the *Kpn-P*_*GAL1*_*-BamHI* fragment with *Kpn-P*_*TEF1*_*-BamHI* fragment, *BamHI-YFP-EcoRI* fragment with *BamHI-mCherry-EcoRI* fragment, in the plasmid pHIS5G1YFP. For XLUYLmCdd80, we knocked out *GAL80* from both MAURA3-YFP and XLLHmC, and mated the resulting strains.

### Growth conditions, media and flow cytometry

Cultures were grown in synthetic complete media. All growths were performed in a 30 °C shaker at 220 rpm with a total volume of 1 mL, except for during the competition period, for ~ 8 h per day growths were performed in 96-well plates (Costar® 3799) in a 30 °C plate shaker at 880 rpm with a total volume of 100 μL. After 48 h of growth on minimal media plates with 2% glucose, 0.03% mannose was used as a non-inducing carbon source for the 22 h overnight growth for all 16 strains and the competing strain. This was followed by a 72 h induction period in one of the six competition environments with different combinations of carbon source concentrations. The six environments are as follows:

**Table:** 

Environment	Galactose Concentration	Mannose Concentration
A	0.03%	0.1%
B	0.1%	0.03%
C	0.1%	0
D	1%	0
E	0.3%	0
F	2%	0

By the end of the induction period, each culture of the 16 strains was mixed with the competing strain to target a final ratio of 1:1 (3:7 for environment A) in terms of population size. OD_600_ of each culture was measured for mixing volume calculation. Right after mixing, the population ratios were verified by flow cytometry and the mixtures were diluted into fresh media of the same environment and grown for another 24 h. At that point, the expression levels of the two populations had reached steady state in the exact same environment and any effect from even slight OD_600_ difference at time of mixing had been eliminated. The population ratios were then measured and recorded as the starting point of the competition period, and were subsequently recorded every 24 h for another 48 h. In all flow cytometry measurements, ~ 25,000 cells were recorded by FACS-Aria (Becton Dickinson) at flow rate 11. An FSC-SSC (forward scatter - side scatter) gate representing the densest 30~ 40% of total population was applied during data analysis to eliminate individuals with unusual morphologies, such as dying cells and cell debris. For each group, no significant day-to-day growth rate variation was observed during the entire experiment. Starting from the beginning of induction, cell densities were kept below OD_600_ 0.15 for environments A and B, and between OD_600_ 0.2 and 0.3 for all other environments to prevent nutrient depletion.

To test the fitness measurement’s invariability to the mixing ratio of the populations, the sixteen strains were also mixed with the competing strain and grown under environment C using the same procedure as described above, except that 3:7 and 7:3 were used as the targeted final ratio instead of 1:1. The fitness values from these experiments are then quantified as usual, and compared with each other and with results from the same experiment performed with a 1:1 target ratio using a two-sample Student’s t-test. The Benjamini-Hochberg procedure [35] was applied to control the false discovery rate at *α* = 0.05.

For the control experiment in 2% glucose, strains were grown on minimal media plates with 2% glucose for 48 h, then inoculated into synthetic complete liquid media with 2% glucose as the sole carbon source for the 22 h overnight growth. This was followed by another 22 h of growth in the same 2% glucose liquid media environment, with a final OD_600_ below 0.15. YFP and mCherry expression levels of each strain were then measured by FACS as described above.

### Quantification of fitness and expression values

The raw expression level of each strain during the experiment is measured by averaging the P_*GAL1*_-YFP fluorescence of the cells as measured by flow cytometry. To control for the effect of different cell density, the raw expression levels are normalized using the average expression level of the reference strain from the same sample. This measurement is performed three times per individual experiment: at the start of the competition period, 24 h after the start of the competition period, and 48 h after the start of the competition period. The average of the three normalized expression levels were taken as the expression level of the strain from that experiment.

The fitness value of each strain relative to the reference strain is calculated from the population ratio of the cells in the sample at the beginning and end of the competition period as depicted in Fig. 1b and then normalized to the average fitness value of the wild-type strain for ease of comparison.

### Quantification of average effect of gene dosage reduction on fitness

In Figs. 2e through 5e as well as Additional file 1: Figures. S3E and S4E, we quantified and plotted the average effect of gene dosage reduction on the fitness of the yeast strain. Without loss of generality, we describe below how we quantified the average effect of halving the dosage of *GAL80*. The same method was separately applied for each gene to obtain the results depicted in the figures.

First, we identified the 8 genetic backgrounds in which the dosage of *GAL80* was halved, which are:

Here, the genetic background of each *S. cerevisiae* strain is specified by a square composed of 4 small squares. The small squares represent the four regulatory genes (blue for *GAL3*, red for *GAL80*, green for *GAL2*, yellow for *GAL4*). The square is fully filled if the dosage of the gene is unchanged from wild type and half-filled if the dosage of the gene is halved. For example, represents the wild type *S. cerevisiae*, while represents a strain with the dosage of all four regulatory genes halved (the “quadruple deletion” strain).

For each growth environment, the fitness values of the strains with *GAL80* dosage halved were subtracted from the fitness values of the strains with *GAL80* dosage unchanged in an otherwise identical genetic background, and these differences were averaged as shown:

The results of this averaging process, performed separately for each environment, were plotted in the figures. The uncertainty in the result is calculated from the s.e.m. of the measured fitness values used to compute the result and plotted as error bars in the figure.

### Quantification of epistatic deviations

Overall and net epistatic deviations are quantified according to the approach in [36]. Briefly, the overall epistatic deviation of a strain with dosage reduction in the gene set *M* is

$$ {\varepsilon}_M={w}_M-{\prod}_{i\in M}{w}_i $$

where *M* is the set of genes with reduced dosage, *w*_*M*_ is the relative fitness of a strain with the dosage of all genes in *M* reduced, and *w*_*i*_ is the relative fitness of a strain with only the dosage of gene *i* reduced. The expected fitness in the absence of epistatic interactions is the product of fitness of the strains that are dosage-reduced in only one gene. The uncertainty in the result is calculated from the s.e.m. of the measured fitness values used to compute the result and plotted as error bars in the figure.

The net epistatic deviation for higher-order epistatic interactions are computed by removing the net effects of all lower-order epistatic interactions:

$$ \varepsilon {\prime}_{AB C}={\varepsilon}_{AB C}-{\varepsilon}_{AB}-{\varepsilon}_{BC}-{\varepsilon}_{AC} $$

$$ \varepsilon {\prime}_{AB C D}={\varepsilon}_{AB C D}-{\varepsilon}_{AB C}^{\prime }-{\varepsilon}_{AB D}^{\prime }-{\varepsilon}_{AC D}^{\prime }-{\varepsilon}_{BC D}^{\prime }-{\varepsilon}_{AB}-{\varepsilon}_{BC}-{\varepsilon}_{AC}-{\varepsilon}_{AD}-{\varepsilon}_{BD}-{\varepsilon}_{CD}={\varepsilon}_{AB C D}-{\varepsilon}_{AB C}-{\varepsilon}_{AB D}-{\varepsilon}_{AC D}-{\varepsilon}_{BC D}+{\varepsilon}_{AB}+{\varepsilon}_{BC}+{\varepsilon}_{AC}+{\varepsilon}_{AD}+{\varepsilon}_{BD}+{\varepsilon}_{CD} $$

Where *A, B, C,* and *D* represents genes whose dosage was reduced. The uncertainty in the result is calculated from the uncertainty in the overall epistatic deviations used to compute the result and plotted as error bars in the figure.

Significance of epistatic deviations are assessed using a two-tailed Z-test (*H*_0_ : *ε* = 0), controlling the family-wise error rate using the Holm-Bonferroni procedure [37].

## Additional file

Additional file 1: **Figure S1.** Expression level distributions of the strains used in this study in a 2% glucose environment. Center: Expression level distributions of the 16 strains under study. Bottom corners: expression level distributions of the two reference strains: left: the *gal80Δ* strain (XLUYLmCdd80); right: the WT strain with P*TEF*-mCherry (XLUYLmC). Glucose catabolite repression and the repression from Gal80p combine to eliminate all expression from the P*GAL1*-YFP reporter in all strains except the *gal80Δ* strain. **Figure S2.** Fitness level measured is invariant to the initial population ratio. Fitness measurements are performed for all 16 strains in Environment D (1% galactose) using different initial fractions: red = 67% (s.d. = 7%), green = 27% (s.d. = 3%), blue = 54% (s.d. = 6%). The results are shown above. Error bars indicate s.e.m. (*N* = 9). No statistically significant differences were observed (using Student’s t-test with the Benjamini–Hochberg procedure to control false discovery rate at 0.05). **Figure S3.** Results of the competition experiment in Environment E (0.3% galactose). **A.** Final expression level distributions of the 16 strains. **B.** Average P*GAL1*-YFP expression level of the 16 strains. Expression levels are normalized to the expression level of the reference strain in the same sample. Error bars indicate s.e.m. (*N* = 9). Stars indicate statistically significant differences from wild-type strain as determined by a two-tailed Student’s t-test (Bonferroni-corrected *p*-value: ****: *p* < 0.0001; ***: *p* < 0.001; **: *p* < 0.01; *: *p* < 0.05). **C.** Average fitness value of the 16 strains, normalized to the average 5 fitness value of the wild-type strain. Error bars indicate s.e.m. (*N* = 9). Stars indicate statistically significant differences from wild-type strain as determined by a two-tailed Student’s t-test (Bonferroni-corrected *p*-value: ****: *p* < 0.0001; ***: *p* < 0.001; **: *p* < 0.01; *: *p* < 0.05). **D.** Plot of expression vs. fitness for the 16 strains. Solid line is the prediction of the fitted linear model. Error bars indicate s.e.m. (*N* = 9). **E**. Average effect of copy number reduction on fitness for the four genes in this environment. Error bars indicate uncertainty calculated from the s.e.m. of the fitness measurements. **Figure S4.** Results of the competition experiment in Environment F (2% galactose). **A.** Final expression level distributions of the 16 strains. **B.** Average P*GAL1*-YFP expression level of the 16 strains. Expression levels are normalized to the expression level of the reference strain in the same sample. Error bars indicate s.e.m. (N = 9). Stars indicate statistically significant differences from wild-type strain as determined by a twotailed Student’s t-test (Bonferroni-corrected p-value: ****: *p* < 0.0001; ***: *p* < 0.001; **: *p* < 0.01; *: *p* < 0.05). **C.** Average fitness value of the 16 strains, normalized to the average fitness 7 value of the wild-type strain. Error bars indicate s.e.m. (N = 9). Stars indicate statistically significant differences from wild-type strain as determined by a two-tailed Student’s t-test (Bonferroni-corrected p-value: ****: *p* < 0.0001; ***: *p* < 0.001; **: *p* < 0.01; *: *p* < 0.05). **D.** Plot of expression vs. fitness for the 16 strains. Solid line is the prediction of the fitted linear model; dotted line shows the prediction of the model at 1% galactose. Error bars indicate s.e.m. (N = 9). **E**. Average effect of copy number reduction on fitness for the four genes in this environment. Error bars indicate uncertainty calculated from the s.e.m. of the fitness measurements. **Figure S5.** Epistasis analysis. **A-F:** Left: Overall epistatic deviation of strains with the dosage of more than one gene reduced for environments A through F, respectively. Right: Net epistatic deviation for higher-order interactions for environments A through F, respectively. Error bars indicate uncertainty calculated from the s.e.m. of the fitness measurements. Stars indicate statistically significant epistatic deviation as determined by a two-sided Z-test, with the family-wise error rate controlled using the Holm- Bonferroni procedure: ****: α = 0.0001; ***: α = 0.001; **: α = 0.01; *: α = 0.05. **Table S1.** *Saccharomyces cerevisiae* strains used in the study. All strains are built on the W303 genetic background. (PDF 1334 kb)
