# Supplementary material for: Multi-component gene network design as a survival strategy in diverse environments
Source: BMC Syst Biol. 2018 Sep 26;12:85. doi: 10.1186/s12918-018-0609-3 (PMC6158886; doi:10.1186/s12918-018-0609-3)
Supplement: Supplementary file 1 — Figure S1. Expression level distributions of the strains used in this study in a 2% glucose environment. Center: Expression level distributions of the 16 strains under study. Bottom corners: expression level distributions of the two reference strains: left: the gal80Δ strain (XLUYLmCdd80); right: the WT strain with PTEF-mCherry (XLUYLmC). Glucose catabolite repression and the repression from Gal80p combine to eliminate all expression from the PGAL1-YFP reporter in all strains except the gal80Δ strain. Figure S2. Fitness level measured is invariant to the initial population ratio. Fitness measurements are performed for all 16 strains in Environment D (1% galactose) using different initial fractions: red = 67% (s.d. = 7%), green = 27% (s.d. = 3%), blue = 54% (s.d. = 6%). The results are shown above. Error bars indicate s.e.m. (N = 9). No statistically significant differences were observed (using Student’s t-test with the Benjamini–Hochberg procedure to control false discovery rate at 0.05). Figure S3. Results of the competition experiment in Environment E (0.3% galactose). A. Final expression level distributions of the 16 strains. B. Average PGAL1-YFP expression level of the 16 strains. Expression levels are normalized to the expression level of the reference strain in the same sample. Error bars indicate s.e.m. (N = 9). Stars indicate statistically significant differences from wild-type strain as determined by a two-tailed Student’s t-test (Bonferroni-corrected p-value: ****: p < 0.0001; ***: p < 0.001; **: p < 0.01; *: p < 0.05). C. Average fitness value of the 16 strains, normalized to the average 5 fitness value of the wild-type strain. Error bars indicate s.e.m. (N = 9). Stars indicate statistically significant differences from wild-type strain as determined by a two-tailed Student’s t-test (Bonferroni-corrected p-value: ****: p < 0.0001; ***: p < 0.001; **: p < 0.01; *: p < 0.05). D. Plot of expression vs. fitness for the 16 strains. Solid line is the prediction [file 12918_2018_609_MOESM1_ESM.pdf]

Multi-component gene network design as a survival strategy in diverse environments

Xinyue Luo[1,2,†], Ruijie Song[2,3,†], and Murat Acar[1,2,3,4,*]

[1] Department of Molecular Cellular and Developmental Biology, Yale University
219 Prospect Street, New Haven, CT 06511

[2] Systems Biology Institute, Yale University
850 West Campus Drive, West Haven, CT 06516

[3] Interdepartmental Program in Computational Biology and Bioinformatics, Yale University
300 George Street, Suite 501, New Haven, CT 06511

[4] Department of Physics, Yale University
217 Prospect Street, New Haven, CT 06511

[†] These authors contributed equally to this work.

* To whom correspondence should be addressed: murat.acar@yale.edu

SUPPLEMENTARY INFORMATION

Supplementary Figures S1-S5

Supplementary Table S1

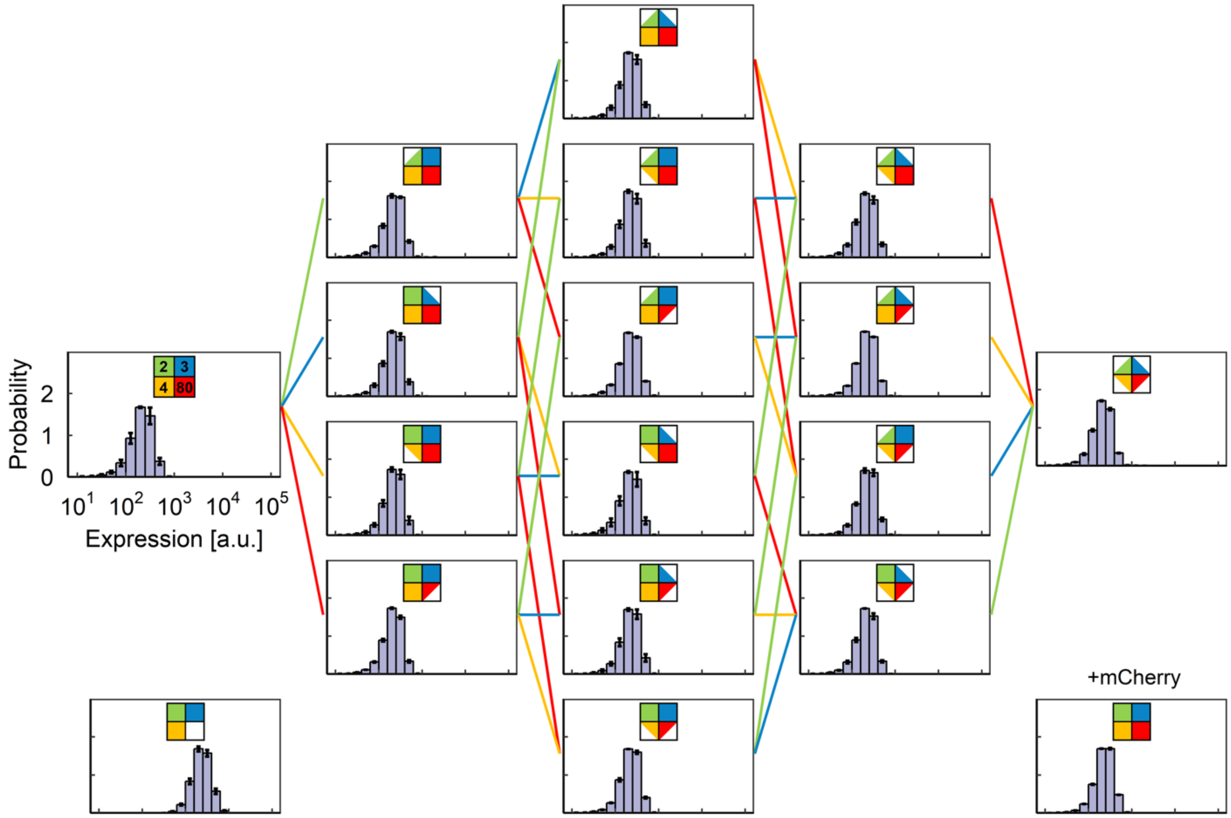

Supplementary Figure S1. Expression level distributions of the strains used in this study in a 2% glucose environment. Center: Expression level distributions of the 16 strains under study. Bottom corners: expression level distributions of the two reference strains: left: the *gal80*Δ strain (XLUYLmCdd80); right: the WT strain with P$_{TEF}$-mCherry (XLUYLmC). Glucose catabolite repression and the repression from Gal80p combine to eliminate all expression from the P$_{GAL1}$-YFP reporter in all strains except the *gal80*Δ strain.

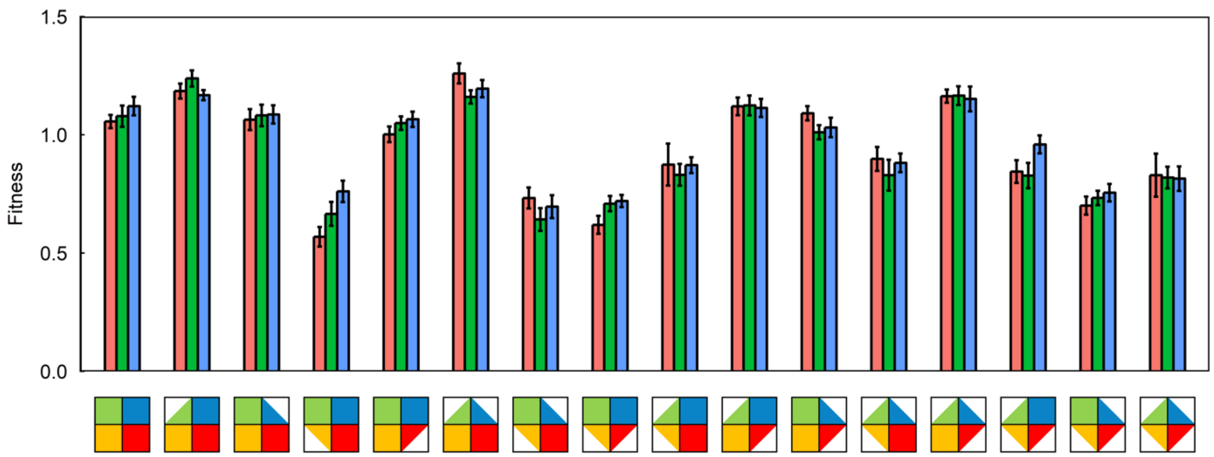

Supplementary Figure S2. Fitness level measured is invariant to the initial population ratio. Fitness measurements are performed for all 16 strains in Environment D (1% galactose) using different initial fractions: red = 67% (s.d. = 7%), green = 27% (s.d. = 3%), blue = 54% (s.d. = 6%). The results are shown above. Error bars indicate s.e.m. (N=9). No statistically significant differences were observed (using Student's t-test with the Benjamini–Hochberg procedure to control false discovery rate at 0.05).

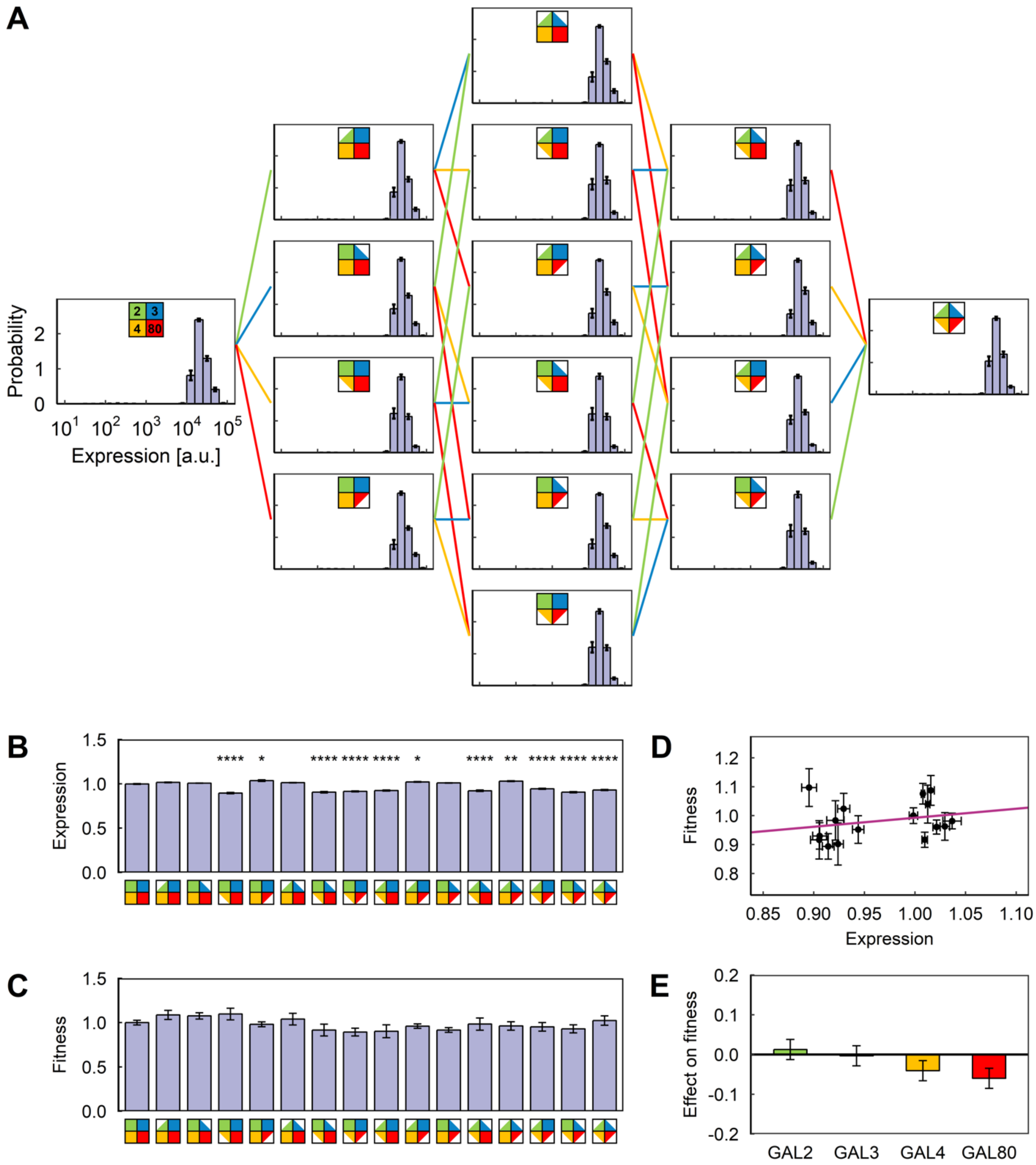

Supplementary Figure S3. Results of the competition experiment in Environment E (0.3% galactose). A. Final expression level distributions of the 16 strains. **B.** Average P_{GAL1}-YFP expression level of the 16 strains. Expression levels are normalized to the expression level of the reference strain in the same sample. Error bars indicate s.e.m. (N=9). Stars indicate statistically significant differences from wild-type strain as determined by a two-tailed Student's t-test (Bonferroni-corrected p-value: ****: $p < 0.0001$; ***: $p < 0.001$; **: $p < 0.01$; *: $p < 0.05$). **C.** Average fitness value of the 16 strains, normalized to the average

fitness value of the wild-type strain. Error bars indicate s.e.m. (N=9). Stars indicate statistically significant differences from wild-type strain as determined by a two-tailed Student's t-test (Bonferroni-corrected p-value: ****: $p < 0.0001$; ***: $p < 0.001$; **: $p < 0.01$; *: $p < 0.05$). **D.** Plot of expression vs. fitness for the 16 strains. Solid line is the prediction of the fitted linear model. Error bars indicate s.e.m. (N=9). **E**. Average effect of copy number reduction on fitness for the four genes in this environment. Error bars indicate uncertainty calculated from the s.e.m. of the fitness measurements.

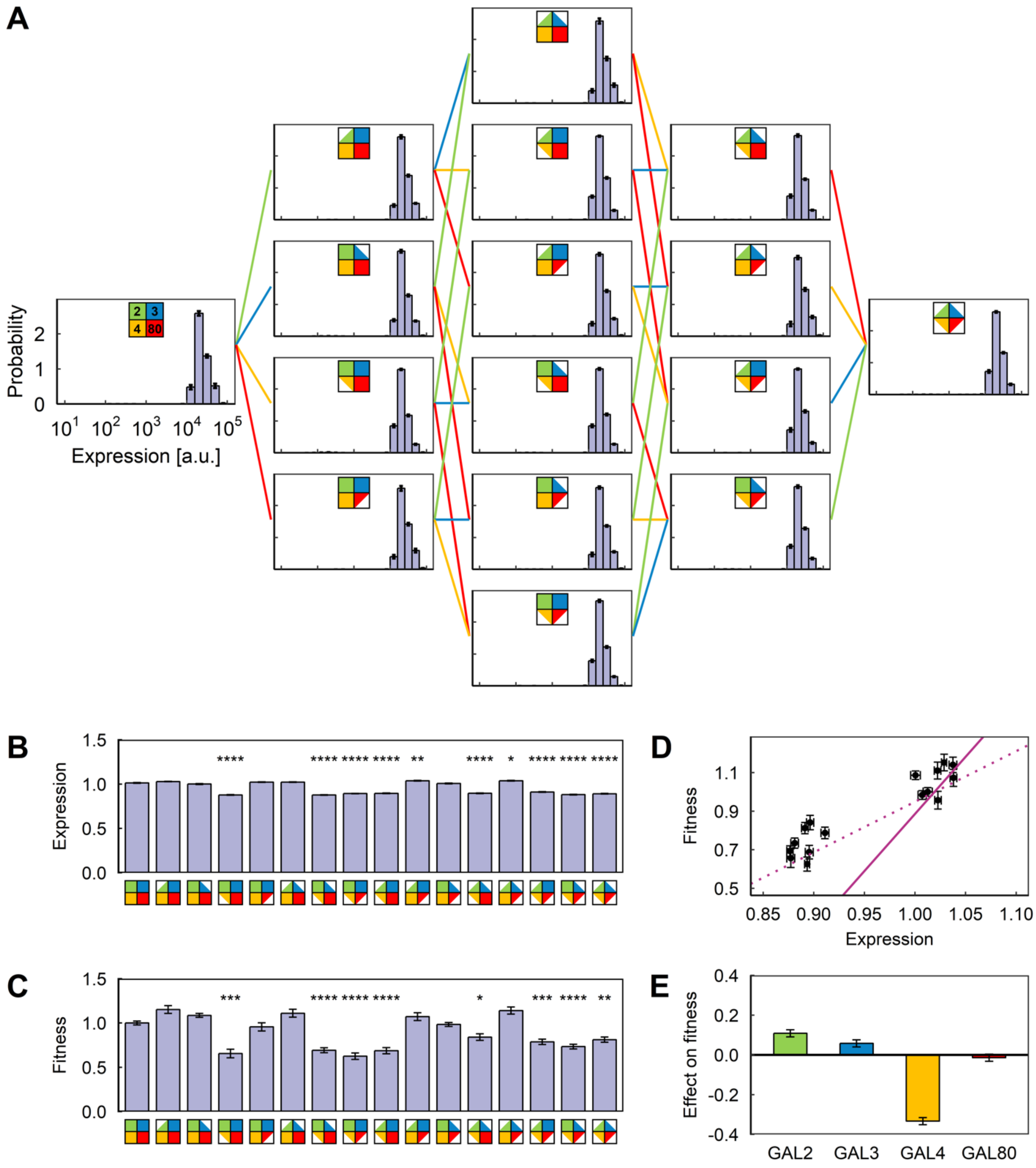

Supplementary Figure S4. Results of the competition experiment in Environment F (2% galactose). A. Final expression level distributions of the 16 strains. **B.** Average P$_{GAL1}$-YFP expression level of the 16 strains. Expression levels are normalized to the expression level of the reference strain in the same sample. Error bars indicate s.e.m. (N=9). Stars indicate statistically significant differences from wild-type strain as determined by a two-tailed Student's t-test (Bonferroni-corrected p-value: ****: p < 0.0001; ***: p < 0.001; **: p < 0.01; *: p < 0.05). **C.** Average fitness value of the 16 strains, normalized to the average fitness

value of the wild-type strain. Error bars indicate s.e.m. (N=9). Stars indicate statistically significant differences from wild-type strain as determined by a two-tailed Student's t-test (Bonferroni-corrected p-value: ****: $p < 0.0001$; ***: $p < 0.001$; **: $p < 0.01$; *: $p < 0.05$). **D.** Plot of expression vs. fitness for the 16 strains. Solid line is the prediction of the fitted linear model; dotted line shows the prediction of the model at 1% galactose. Error bars indicate s.e.m. (N=9). **E**. Average effect of copy number reduction on fitness for the four genes in this environment. Error bars indicate uncertainty calculated from the s.e.m. of the fitness measurements.

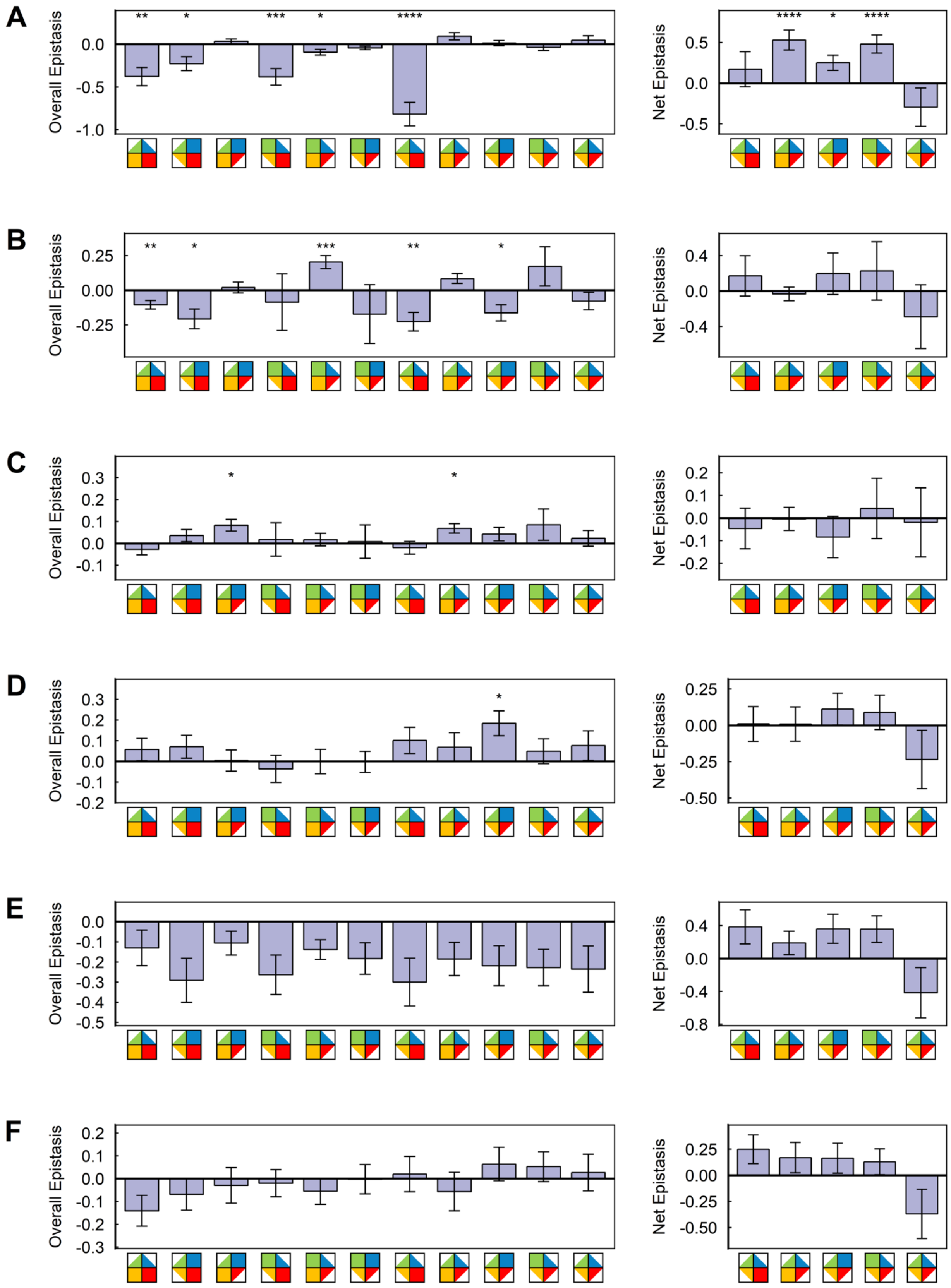
A
B
C
D
E
F
Overall Epistasis
Net Epistasis
*
**
***
****

Supplementary Figure S5. Epistasis analysis. A-F: Left: Overall epistatic deviation of strains with the dosage of more than one gene reduced for environments A through F, respectively. Right: Net epistatic deviation for higher-order interactions for environments A through F, respectively. Error bars indicate uncertainty calculated from the s.e.m. of the fitness measurements. Stars indicate statistically significant epistatic deviation as determined by a two-sided Z-test, with the family-wise error rate controlled using the Holm-Bonferroni procedure: ****: $\alpha = 0.0001$; ***: $\alpha = 0.001$; **: $\alpha = 0.01$; *: $\alpha = 0.05$.

Strain	Genotype
MA0001	*MATα*
MA0002	*MATa*
MAURA3-YFP	*MATα, ho::HIS5-P*$_{GAL1}$*-YFP, ura3::URA3*
MALEU2	*MATa, leu2::LEU2*
XLUYgal2KO	*MATα, ho::HIS5-P*$_{GAL1}$*-YFP, ura3::URA3, gal2Δ::KanMX4*
XLUYgal3KO	*MATα, ho::HIS5-P*$_{GAL1}$*-YFP, ura3::URA3, gal3Δ::NatNT2*
XLUYΔ2Δ3	*MATα, ho::HIS5-P*$_{GAL1}$*-YFP, ura3::URA3, gal2Δ::KanMX4, gal3Δ::NatNT2*
XLLgal4KO	*MATa, leu2::LEU2, gal4Δ::KanMX4*
XLLgal80KO	*MATa, leu2::LEU2, gal80Δ::NatNT2*
XLUYΔ4Δ80	*MATa, leu2::LEU2, gal4Δ::KanMX4, gal80Δ::NatNT2*
XLLHmC	*MATa, ho::HIS5-P*$_{TEF1}$*-mCherry, leu2::LEU2*
XLUYLΔ2	*MATa/α, ho/ho::HIS5-P*$_{GAL1}$*-YFP, ura3/ura3::URA3, leu2::LEU2/leu2, GAL2/gal2Δ::KanMX4*
XLUYLΔ3	*MATa/α, ho/ho::HIS5-P*$_{GAL1}$*-YFP, ura3/ura3::URA3, leu2::LEU2/leu2, GAL3/gal3Δ::NatNT2*
XLUYLΔ4	*MATa/α, ho/ho::HIS5-P*$_{GAL1}$*-YFP, ura3/ura3::URA3, leu2::LEU2/leu2, gal4Δ::KanMX4/GAL4*
XLUYLΔ80	*MATa/α, ho/ho::HIS5-P*$_{GAL1}$*-YFP, ura3/ura3::URA3, leu2::LEU2/leu2, gal80Δ::NatNT2/GAL80*
XLUYLΔ23	*MATa/α, ho/ho::HIS5-P*$_{GAL1}$*-YFP, ura3/ura3::URA3, leu2::LEU2/leu2, GAL2/gal2Δ::KanMX4, GAL3/gal3Δ::NatNT2*
XLUYLΔ24	*MATa/α, ho/ho::HIS5-P*$_{GAL1}$*-YFP, ura3/ura3::URA3, leu2::LEU2/leu2, GAL2/gal2Δ::KanMX4, gal4Δ::KanMX4/GAL4*
XLUYLΔ28	*MATa/α, ho/ho::HIS5-P*$_{GAL1}$*-YFP, ura3/ura3::URA3, leu2::LEU2/leu2, GAL2/gal2Δ::KanMX4, gal80Δ::NatNT2/GAL80*
XLUYLΔ34	*MATa/α, ho/ho::HIS5-P*$_{GAL1}$*-YFP, ura3/ura3::URA3, leu2::LEU2/leu2, GAL3/gal3Δ::NatNT2, gal4Δ::KanMX4/GAL4*
XLUYLΔ38	*MATa/α, ho/ho::HIS5-P*$_{GAL1}$*-YFP, ura3/ura3::URA3, leu2::LEU2/leu2, GAL3/gal3Δ::NatNT2, gal80Δ::NatNT2/GAL80*

XLUYLΔ48	*MATa/α, ho/ho::HIS5-P_{GAL1}-YFP, ura3/ura3::URA3, leu2::LEU2/leu2, gal4Δ::KanMX4/GAL4, gal80Δ::NatNT2/GAL80*
XLUYLΔ234	*MATa/α, ho/ho::HIS5-P_{GAL1}-YFP, ura3/ura3::URA3, leu2::LEU2/leu2, GAL2/gal2Δ::KanMX4, GAL3/gal3Δ::NatNT2, gal4Δ::KanMX4/GAL4*
XLUYLΔ348	*MATa/α, ho/ho::HIS5-P_{GAL1}-YFP, ura3/ura3::URA3, leu2::LEU2/leu2, GAL3/gal3Δ::NatNT2, gal4Δ::KanMX4/GAL4, gal80Δ::NatNT2/GAL80*
XLUYLΔ238	*MATa/α, ho/ho::HIS5-P_{GAL1}-YFP, ura3/ura3::URA3, leu2::LEU2/leu2, GAL2/gal2Δ::KanMX4, GAL3/gal3Δ::NatNT2, gal80Δ::NatNT2/GAL80*
XLUYLΔ248	*MATa/α, ho/ho::HIS5-P_{GAL1}-YFP, ura3/ura3::URA3, leu2::LEU2/leu2, GAL2/gal2Δ::KanMX4, gal4Δ::KanMX4/GAL4, gal80Δ::NatNT2/GAL80*
XLUYLq	*MATa/α, ho/ho::HIS5-P_{GAL1}-YFP, ura3/ura3::URA3, leu2::LEU2/leu2, GAL2/gal2Δ::KanMX4, GAL3/gal3Δ::NatNT2, gal4Δ::KanMX4/GAL4, gal80Δ::NatNT2/GAL80*
XLUYLw	*MATa/α, ho/ho::HIS5-P_{GAL1}-YFP, ura3/ura3::URA3, leu2::LEU2/leu2*
XLUYLmC	*MATa/α, ho::HIS5-P_{TEF1}-mCherry/ho::HIS5-P_{GAL1}-YFP, ura3/ura3::URA3, leu2::LEU2/leu2*
XLUYLmCdd80	*MATa/α, ho::HIS5-P_{TEF1}-mCherry/ho::HIS5-P_{GAL1}-YFP, ura3/ura3::URA3, leu2::LEU2/leu2, gal80Δ::NatNT2/gal80Δ::NatNT2*

Supplementary Table S1. *Saccharomyces cerevisiae* strains used in the study. All strains are built on the W303 genetic background.
